# Metabolomic profiling of developing perilla leaves reveals the best harvest time

**DOI:** 10.3389/fpls.2022.989755

**Published:** 2022-11-30

**Authors:** Jiabao Chen, Long Guo, Guiya Yang, Aitong Yang, Yuguang Zheng, Lei Wang

**Affiliations:** ^1^ College of Pharmacy, Hebei University of Chinese Medicine, Shijiazhuang, China; ^2^ Traditional Chinese Medicine Processing Technology Innovation Center of Hebei Province, School of Pharmacy, Hebei University of Chinese Medicine, Shijiazhuang, China; ^3^ International Joint Research Center on Resource Utilization and Quality Evaluation of Traditional Chinese Medicine of Hebei Province, School of Pharmacy, Hebei University of Chinese Medicine, Shijiazhuang, China; ^4^ Department of Pharmaceutical Engineering, Hebei Chemical and Pharmaceutical College, Shijiazhuang, China

**Keywords:** Perilla leaf, mass spectrometry, metabolomic dynamics, harvest time, multivariate statistical analysis

## Abstract

Ultra-performance liquid chromatography-tandem mass spectrometry (UPLC-MS) and gas chromatography-mass spectrometry (GC-MS) were applied to analyze metabolites in perilla leaves (PLs) during its developmental process. In total, 118 metabolites were identified, including volatile and non-volatile compounds, such as terpenoids, sugars, amino acids, organic acids, fatty acids, phenolic acids, flavonoids, and others. Principal component analysis (PCA) indicated great variations of metabolites during PLs development. Clustering analysis (CA) clarified the dynamic patterns of the metabolites. The heatmap of CA showed that most of the detected metabolites were significantly accumulated at stage 4 which is the pre anthesis period, and declined afterwards. The results of the present study provide a comprehensive overview of the metabolic dynamics of developing PLs which suggested that pre anthesis period is the best harvest time for PLs.

## 1 Introduction

*Perilla frutescens* (L.) Britt. is an annual herbal plant that belongs to the family of Lamiaceae. It is widely cultivated in Asia counties, such as China, Japan, Korea, Vietnam and other regions (Yu et al., 2017; Zhang et al., 2021). Perilla leaves (PLs) are commonly consumed as kitchen herb in salads, sushi, soups, and as spice, garnish, or food colorant. PLs are also used as traditional Chinese medicine to relieve exterior, dispersing cold, ease stomach pain, reduce phlegm and relieve cough and asthma (Ha et al., 2012; Igarashi and Miyazaki, 2013). Phytochemical studies indicated PLs were rich in essential oils, flavonoids, fatty acids, phenolic compounds, etc (Ahmed, 2018). Compounds of PLs showed various biological activities such as antioxidant, antimicrobial, anti-allergic, antidepressant, anti-inflammatory, and anticancer effects (Banno et al., 2004; Ghimire et al., 2019; Wang et al., 2021; Yang et al., 2021). PLs has been used as a natural herbal medicine for treatment of depression-related disease, asthma, tumors, coughs, allergies, intoxication, fever, chills, headache, stuffy nose, and some intestinal disorders (Ito et al., 2011; Kim et al., 2012; Zhou et al., 2021). Owing to these health benefits, the food and pharmaceutical industries are increasingly interested in PLs.

The pharmacological activities of perilla are closely related to its chemical constituents. Some studies have revealed that great dynamic variation in the nutritional components and phytochemical substances might occur during plant development. Ghimire et al. (Ghimire et al., 2017) compared the total volatile contents of eighteen accessions of PLs and most of them were higher before the flowering time than at the flowering stage. Luo et al. (Luo et al., 2021) invested variation of two phenolic acids and six flavonoids during PLs development and suggested to harvest PLs at different times basing on the targeted metabolites. Peiretti et al. (Peiretti, 2011) evaluated perilla quality according to the content of fatty acid, fiber, crude protein, organic matter and gross energy during the growth cycle of perilla. According to their result, it is better to harvest perilla at around two months after sowing. Though these studies provided a general feature of perilla nutritional contents, a more comprehensive and detailed dynamic profile of developing PLs is still essential for providing more information to determine the harvest time according to different application.

In this study, mass spectrometry (MS) based high throughput metabolomic platforms were applied to ascertain the dynamic trajectory of complex ingredients of PLs during developmental process. In addition, multiple statistical analysis methods, including principal component analysis (PCA) and Clustering analysis (CA) were used to clarify the dynamic patterns of the detected metabolites. These data provide data support for determining the best harvest time of perilla leaves.

## 2 Materials and methods

### 2.1 Chemicals and reagents

HPLC grade methanol (MeOH), acetonitrile (ACN) and formic acid were purchased from Fisher Scientific (Pittsburgh, PA, United States) Ultrapure water was prepared by Synergy water purification system (Millipore, Billerica, United States). The reserpine standards (HPLC grade) and GC grade derivatizing regent MSTFA (N-methyl-N-(trimethylsilyl)trifluoroacetamide), methoxyamine hydrochloride were purchased from Sigma-Aldrich (St. Louis, MO, USA). Chemical reagent n-hexane (GC grade) and Anhydrous pyridine (GC grade) were obtained from Shanghai Aladdin Biochemical Technology Co., Ltd. (Shanghai, China). Salicylic acid, luteolin, apigenin and rosmarinic acid standards were provided by Shanghai Yuanye Bio-Technology Co., Ltd. (Shanghai, China). Reference standards of luteolin-7-O-glucoside, scutellarin, luteolin-7-O-glucuronide, apigenin-7-glucoside and apigenin-7-O-glucuronide were purchased from Shanghai Standard Technology Co., Ltd. (Shanghai, China). The purities of all standards were determined to be higher than 98%. Other chemicals and reagents were analytical grade.

### 2.2 Plant materials

The PLs were randomly collected from *Perilla frutescens* (L.) Britt. cultivated in the plant base of Hebei Academy of Agriculture and Forestry Sciences in Shijiazhuang (China 38°06′41.7′′ N, 114°45′35.8′′E) in mid May 2019, and the samples were collected semimonthly from July 2019 to October 2019. The mean annual temperature was 14.4°C, mean annual humidity was 62%, mean annual precipitation was 422.6 mm, mean annual sunshine hours was 2235.4 hours. Growth process of perilla was performed using manual fertilization, therefore, soil is rich in organic elements. Three biological replicates were collected for each developmental phase (**Table 1**). The plant was identified by professor Yuguang Zheng (Hebei Chemical and Pharmaceutical College, China), and voucher specimens were deposited in Traditional Chinese Medicine Processing Technology Innovation Center of Hebei Province, Hebei University of Chinese Medicine. The harvested leaves were air-dried in the dark at room temperature for 2 weeks to acquire consistently low water content.

**Table 1:** Information of samples collected at different developmental times.

No.	Collection date	Growth phase (Wei et al., 2017)	Sample number	Specimen No.
1	July 15, 2019	nutritional phase	stage 1-1	PF2019071501
2	July 15, 2019	nutritional phase	stage 1-2	PF2019071502
3	July 15, 2019	nutritional phase	stage 1-3	PF2019071503
4	July 30, 2019	nutritional phase	stage 2-1	PF2019073001
5	July 30, 2019	nutritional phase	stage 2-2	PF2019073002
6	July 30, 2019	nutritional phase	stage 2-3	PF2019073003
7	August 15, 2019	nutritional phase	stage 3-1	PF2019081501
8	August 15, 2019	nutritional phase	stage 3-2	PF2019081502
9	August 15, 2019	nutritional phase	stage 3-3	PF2019081503
10	August 30, 2019	nutritional phase	stage 4-1	PF2019083001
11	August 30, 2019	nutritional phase	stage 4-2	PF2019083002
12	August 30, 2019	nutritional phase	stage 4-3	PF2019083003
13	September 15, 2019	flowering phase	stage 5-1	PF2019091501
14	September 15, 2019	flowering phase	stage 5-2	PF2019091502
15	September 15, 2019	flowering phase	stage 5-3	PF2019091503
16	September 30, 2019	flowering phase	stage 6-1	PF2019093001
17	September 30, 2019	flowering phase	stage 6-2	PF2019093002
18	September 30, 2019	flowering phase	stage 6-3	PF2019093003
19	October 15, 2019	fruiting phase	stage 7-1	PF2019101501
20	October 15, 2019	fruiting phase	stage 7-2	PF2019101502
21	October 15, 2019	fruiting phase	stage 7-3	PF2019101503

### 2.3 Analysis of the volatile metabolites by GC-MS

#### 2.3.1 Sample pretreatment

The dried PF samples were pulverized with grinder (FW100, Taisite, Tianjin, China), and screened through 60 mesh sieves. 100 mg of each accurately weighted pulverized sample were thoroughly mixed with 1 mL of n-hexane then sonicated (300 W, 40 kHz) 15 min at room temperature. The extracted solution was centrifuged at 13000 rpm at room temperature for 10 min. The supernatant was injected into the GC-MS for analysis.

#### 2.3.2 Instrument parameters

The GC-MS analysis was performed with an Agilent 7890B-5977B GC-MS (Agilent, Santa Clara, CA, USA) coupled with a HP-5MS capillary column (30 m × 0.25 mm, 0.25 μm film thickness, Agilent, Santa Clara, CA, USA). Helium (≥ 99.999%) was used as carrier gas at a constant flow rate of 1.0 mL·min^-1^. 1 μL of the prepared supernatant solution was injected in split-mode with the split ratio set to 2:1 at a temperature of 250°C. The oven temperature program was initially set at 45°C, then increased to 100°C at a rate of 10°C·min^-1^, and subsequently increased to 280°C at a rate of 4°C·min^-1^, finally held for 10 min. The electronic ionization voltage of electron-impact (EI) ion source was 70 eV. The mass spectrometer was operated in full scan mode with a scanning range of 50-550 m/z. n-Alkane standard solution (C8-C20, 40 mg·L^-1^, Sigma-Aldrich, Switzerland) was analyzed under the same condition for retention index (RI) calculation.

### 2.4 Analysis of non-volatile metabolites by GC-MS

#### 2.4.1 Sample pretreatment

An integrative extraction of primary metabolites and secondary metabolites was performed according to a universal extraction protocol (Weckwerth et al., 2010; Mari et al., 2013; Wang et al., 2017) with some modifications. 100 mg of each pulverized samples were extracted with 1 mL of extraction solution (methanol: water: formic acid = 70:28:2) by sonication 15 min. The crude extract was centrifuged at 13,000 rpm at room temperature for 10 min. 50 μL of the supernatant together with 20 μL of salicylic acid (1 mg·mL^-1^, internal standard) was dried using a SpeedVac (Thermo Scientific, Inc., Bremen, Germany) at 5000 rpm and 40°C for 90 min. Methoxyamination of the carbonyl groups was performed by adding 20 μL of methoxyamine hydrochloride (40 mg·mL^-1^) in pyridine to each sample followed by incubation in metal bath at 30°C for 90 min. Subsequently, 80 μL of MSTFA (N-Methyl-N-(trimethylsilyl) trifluoroacetamide) was added and the mixtures were incubated at 37°C for 30 min. The derivatized samples were centrifuged at 13,000 rpm at room temperature for 10 min with the supernatants prepared for GC-MS analysis.

#### 2.4.2 Instrument parameters

Aforementioned GC-MS instrument and column (see 2.3.2) was also applied for analysis of derivatized samples. 1 μL of the derivatized sample was injected using 5:1 split-mode at a temperature of 250°C. The temperature gradient program was as follows: Initial temperature was 80°C, increased to 200°C at a rate of 10°C·min^-1^; then increased to 250°C at a rate of 6°C·min^-1^; subsequently increased to 310°C at a rate of 6°C·min^-1^ and hold at 310°C for 5 min. EI ion source was adjusted to 230°C with electronic energy of 70 eV. The mass spectrometer was determined by the full-scan method ranging from 50 to 550 (m/z). n-Alkane standard solution (C8-C20, 40 mg·L^-1^, Sigma-Aldrich, Switzerland) was analyzed under the same condition for retention index (RI) calculation.

### 2.5 Analysis of the non-volatile metabolites by LC-MS

#### 2.5.1 Sample pretreatment

100 μL of the above mentioned crude extract (see in 2.4.1) was mixed with 100 μL of 5 μg·mL^-1^ reserpine (internal standard) and diluted with 800 μL of extract solution then centrifuged at 13,000 rpm at room temperature for 10 min with the supernatants prepared for the LC-MS analysis.

#### 2.5.2 Instrument parameters

The UHPLC-Q/TOF-MS analysis was performed on an Agilent 1290 UHPLC system coupled with an Agilent 6545 quadrupole time-of-flight mass spectrometer system (Agilent, Santa Clara, CA, United States). Chromatographic separation was performed on an Agilent ZORBAX SB C18 column (4.6 × 50 mm, 1.8 μm).

UHPLC chromatographic conditions: the 0.5 μL of prepared samples were loaded on an Agilent 1290 UHPLC system and eluted with 0.1% formic-water (mobile phase A) and acetonitrile (mobile phase B) in the following gradient: 0-2 min, 12% B; 2-26 min, 12%-24% B; 26-35 min, 24%-50% B; 35-38 min, 50%-100% B; 38-45 min, 100% B. The flow rate was maintained at 0.4 mL·min^-1^, the column temperature was set at 25°C.

The MS acquisition parameters were referred to Chang et al. (2021) with minor modifications. The capillary voltage was set to 4000 V; and the collision energy was 20 eV and 35 eV. The analysis was operated in positive mode with the mass range of m/z 50-1000 Da.

### 2.6 Data processing and multivariate statistical analysis

For qualitative analysis, the metabolites detected by GC-MS with a similarity more than 80% to the NIST17 standard library were identified using the Agilent MassHunter analysis program (Agilent, Santa Clara, CA, USA). The RI of all the identified compounds were calculated by comparing their corresponding peak retention time to that of n-alkanes (C8–C20) (Chaturvedula and Prakash, 2013; Ma et al., 2014). The identification of detected metabolites in the LC-MS analysis was based on their accurate precursor masses and fragment masses. For quantitative analysis, the integrated peak area was considered to be a variable for analysis and normalized to internal standard. The combined GC-MS and LC-MS dataset was transformed to -1~1 by Min-Max Normalization method. SIMCA P13 software (Umetrics, Umea, Sweden) was used for principal component analysis (PCA). Cluster analysis (CA) and heatmap was performed with Origin Pro 2020 (OriginLab Corporation, USA) software. Duncan’s test was performed with IBM SPSS Statistics 23.0 (IBM, USA) software.

## 3 Results and discussion

### 3.1 Identification of detected metabolites

**Figure 1:**
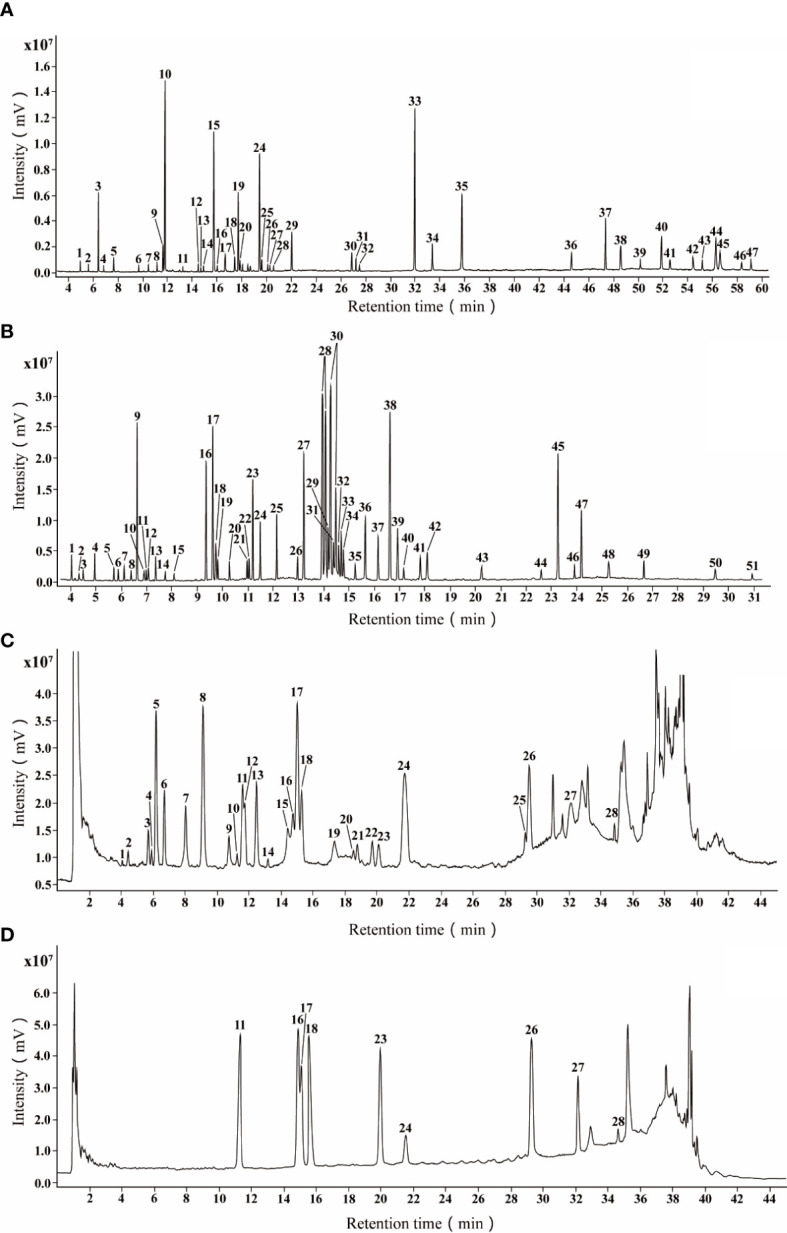
The typical total ion chromatograms of PLs by GC-MS and LC-MS. **(A)** TIC of volatile metabolites in pooled samples by GC-MS; **(B)** TIC of non-volatile metabolites in pooled samples by GC-MS after derivatization; **(C)** TIC of non-volatile metabolites in pooled samples by LC-MS; **(D)** TIC of reference substances by LC-MS.

**Figure 2:**
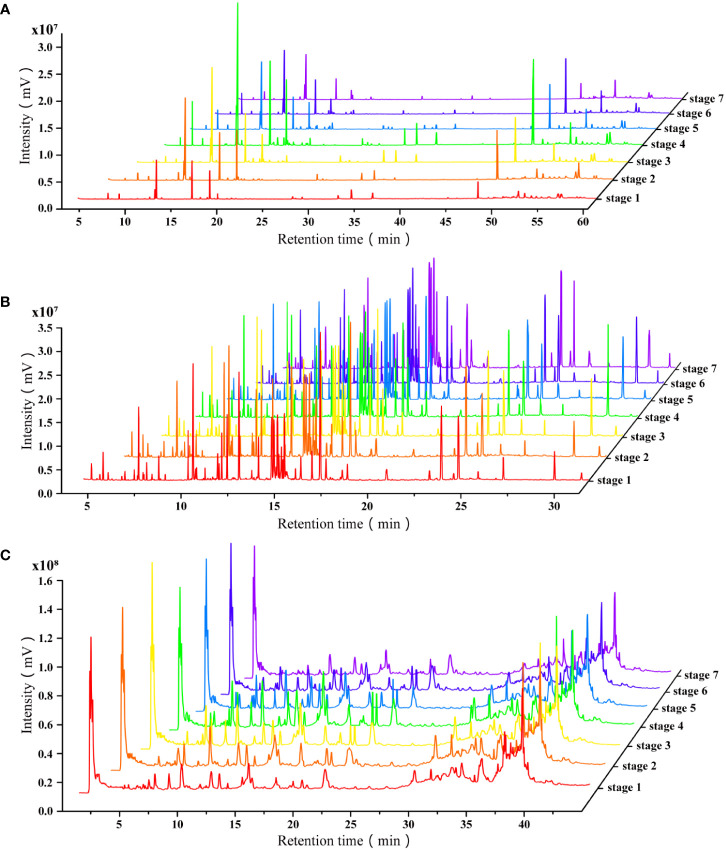
The chemical fingerprints of PLs at different harvesting by GC-MS and LC-MS. **(A)** Fingerprints of volatile metabolites by GC-MS; **(B)** Fingerprints of non-volatile metabolites by GC-MS after derivatization; **(C)** Fingerprints of non-volatile metabolites by LC-MS.

The typical total ion chromatograms (TICs) of GC-MS, pre-column derivatized GC-MS and LC-MS showed metabolomic profiles of PLs (**Figures 1A-C**). With reference to the NIST17 database, 47 volatile metabolites including aldehydes, ketones, alcohols, fatty acids, steroids and others (**Table 2**) were identified according to their retention times and mass spectrums. 51 peaks in **Figure 1B** were identified including sugars, amino acids, organic acids, fatty acids, and phenolic compounds (**Table 3**). The identification of non-volatile metabolites form LC-MS data were based on their precursor ions and fragmentation patterns. 28 metabolites, mainly flavonoids and anthocyanidins, were identified with their detail information such as retention time, chemical formula, ppm errors and fragment ions were listed in **Table 4**. Among the putatively identified compounds, eight metabolites (luteoloside (peak C11), scutellarin (peak C16), luteolin-7-O-glucuronide (peak C17), apigenin-7-O-glucoside (peak C18) apigenin-7-O-glucuronide (peak C23), rosmarinic acid (peak C24), luteolin (peak C26), apigenin (peak C27)) were confirmed with reference substances (**Figure 1D**). The chemical fingerprints showed distinct differences in the chemical composition of PLs at different harvesting (**Figure 2**).

**Table 2:** Identification of volatile compounds analyzed by GC-MS.

No.	RT (min)	Compounds	MF	MW	Class	RI
A1	5.01	α-Pinene	C_10_H_16_	136	Bicyclic monoterpenoids	918
A2	5.65	Pseudolimonene	C_10_H_16_	136	Mononcyclic monoterpenoids	964
A3	6.44	D-Limonene	C_10_H_16_	136	Mononcyclic monoterpenoids	1017
A4	7.51	α-Terpinene	C_10_H_16_	136	Mononcyclic monoterpenoids	1082
A5	7.69	Linalool	C_10_H_18_O	154	Acyclic monoterpenoids	1093
A6	9.71	α-Terpineol	C_10_H_18_O	154	Mononcyclic monoterpenoids	1193
A7	10.53	Nerol	C_10_H_18_O	154	Acyclic monoterpenoids	1228
A8	11.21	Perilla ketone	C_10_H_14_O_2_	166	Acyclic monoterpenoids	1257
A9	11.71	Shisool	C_10_H_18_O	154	Mononcyclic monoterpenoids	1277
A10	11.86	Perillaldehyde	C_10_H_14_O	150	Mononcyclic monoterpenoids	1283
A11	13.43	γ-Elemene	C_15_H_24_	204	Mononcyclic sesquiterpenoids	1344
A12	14.51	α-Copaene	C_15_H_24_	204	Tricyclic sesquiterpenoids	1385
A13	14.76	β-Bourbonene	C_15_H_24_	204	Tricyclic sesquiterpenoids	1394
A14	14.93	β-Elemene	C_15_H_24_	204	Mononcyclic sesquiterpenoids	1401
A15	15.77	β-Caryophyllene	C_15_H_24_	204	Bicyclic sesquiterpenoids	1431
A16	16.16	Perillic acid	C_10_H_14_O_2_	166	Mononcyclic monoterpenoids	1446
A17	16.68	α-Humulene	C_15_H_24_	204	Mononcyclic sesquiterpenoids	1465
A18	17.44	β-Copaene	C_15_H_24_	204	Tricyclic sesquiterpenoids	1492
A19	17.73	Cis-α-Bergamotene	C_15_H_24_	204	Bicyclic sesquiterpenoids	1503
A20	17.87	Bicyclogermacrene	C_15_H_24_	204	Bicyclic sesquiterpenoids	1508
A21	18.09	α-Farnesene	C_15_H_24_	204	Acyclic sesquiterpenoids	1516
A22	18.51	Myristicin	C_11_H_12_O_3_	192	Aromatic compounds	1531
A23	18.59	δ-Cadinene	C_15_H_24_	204	Bicyclic sesquiterpenoids	1534
A24	19.43	Elemicin	C_12_H_16_O_3_	208	Aromatic compounds	1565
A25	19.63	Nerolidol	C_15_H_26_O	222	Acyclic sesquiterpenoids	1572
A26	20.11	Espatulenol	C_15_H_24_O	220	Tricyclic sesquiterpenoids	1590
A27	20.27	β-Caryophyllene oxide	C_15_H_24_O	220	Bicyclic sesquiterpenoids	1595
A28	20.59	α-Patchoulene	C_15_H_24_	204	Tricyclic sesquiterpenoids	1607
A29	22.16	Isoelemicin	C_12_H_16_O_3_	208	Aromatic compounds	1666
A30	26.89	Phytyl acetate	C_22_H_42_O_2_	338	Acyclic diterpenoids	1849
A31	27.23	Pentadecanone	C_18_H_36_O	268	Acyclic sesquiterpenoids	1862
A32	27.52	Myristic acid	C_14_H_28_O_2_	228	Aliphatic compounds	1874
A33	31.96	Palmitic acid	C_16_H_32_O_2_	256	Aliphatic compounds	2059
A34	33.38	Phytol	C_20_H_40_O	296	Acyclic diterpenoids	2118
A35	35.75	α-Linolenic acid	C_18_H_30_O_2_	278	Aliphatic compounds	2217
A36	45.07	Heptacosane	C_27_H_56_	380	Aliphatic compounds	2607
A37	47.41	Squalene	C_30_H_50_	410	Acyclic triterpenoids	2705
A38	48.55	Nonacosane	C_29_H_60_	408	Aliphatic compounds	2753
A39	49.16	1-Heptatriacotanol	C_37_H_76_O	536	Aliphatic compounds	2779
A40	51.90	Hentriacontane	C_31_H_64_	436	Aliphatic compounds	2893
A41	52.60	α-Tocopherol	C_29_H_50_O_2_	430	Mononcyclic triterpenoids	2923
A42	54.44	Campesterol	C_28_H_48_O	400	Steroids	2999
A43	55.19	β-Stigmasterol	C_29_H_48_O	412	Steroids	3031
A44	56.32	Dotriacontane	C_32_H_66_	450	Aliphatic compounds	3078
A45	56.67	γ-Sitosterol	C_29_H_50_O	414	Steroids	3093
A46	58.29	β-Amyrone	C_30_H_48_O	424	Tetracyclic triterpenoids	3161
A47	58.69	α-Amyrin	C_30_H_50_O	426	Tetracyclic triterpenoids	3178

RT, retention time.

MF, molecular formula.

MW, molecular weight.

RI, retention index.

**Table 3:** Identification of non-volatile metabolites analyzed by pre-column derivatization combining with GC-MS.

No.	RT	Compounds	MF	MW	RI
B1	4.03	Lactic acid (2TMS)	C_9_H_22_O_3_Si_2_	234	1063
B2	4.49	L-Alanine (2TMS)	C_9_H_23_NO_2_Si_2_	233	1102
B3	4.67	Glycine (TMS)	C_8_H_21_NO_2_Si_2_	219	1118
B4	4.94	Oxalic acid (2TMS)	C_8_H_18_O_4_Si_2_	234	1141
B5	5.71	Propanedioic acid (2TMS)	C_9_H_20_O_4_Si_2_	248	1206
B6	5.88	L-Valine (2TMS)	C_11_H_27_NO_2_Si_2_	261	1220
B7	6.38	L-Serine (2TMS)	C_9_H_23_NO_3_Si_2_	249	1260
B8	6.57	L-Leucine (2TMS)	C_12_H_29_NO_2_Si_2_	275	1275
B9	6.62	Glycerol (3TMS)	C_12_H_32_O_3_Si_3_	308	1280
B10	6.86	L-Isoleucine (TMS)	C_12_H_29_NO_2_Si_2_	275	1299
B11	6.91	L-Proline (2TMS)	C_11_H_25_NO_2_Si_2_	259	1303
B12	7.03	Glycine (3TMS)	C_11_H_29_NO_2_Si_3_	291	1312
B13	7.36	Glyceric acid (3TMS)	C_12_H_30_O_4_Si_3_	322	1339
B14	7.73	L-Serine (3TMS)	C_12_H_31_NO_3_Si_3_	321	1368
B15	8.08	L-Threonine (3TMS)	C_13_H_33_NO_3_Si_3_	335	1396
B16	9.35	Malic acid (3TMS)	C_13_H_30_O_5_Si_3_	350	1499
B17	9.61	Salicylic acid (2TMS)	C_13_H_22_O_3_Si_2_	282	1522
B18	9.73	L-Aspartic acid (3TMS)	C_13_H_31_NO_4_Si_3_	349	1532
B19	9.81	γ-Aminobutanoic acid (3TMS)	C_13_H_33_NO_2_Si_3_	319	1539
B20	10.27	L-Glutamic acid (3TMS)	C_14_H_33_NO_4_Si_3_	363	1578
B21	10.97	L-Phenylalanine (2TMS)	C_15_H_27_NO_2_Si_2_	309	1639
B22	11.05	L-Asparagine (4TMS)	C_16_H_40_N_2_O_3_Si_4_	420	1646
B23	11.19	Tartaric acid (4TMS)	C_16_H_38_O_6_Si_4_	438	1659
B24	11.48	L-Asparagine (3TMS)	C_13_H_32_N_2_O_3_Si_3_	348	1685
B25	12.14	Xylitol (5TMS)	C_20_H_52_O_5_Si_5_	512	1745
B26	12.84	L-Glutamine (3TMS)	C_14_H_34_N_2_O_3_Si_3_	362	1810
B27	13.21	Citric acid (4TMS)	C_18_H_40_O_7_Si_4_	480	1843
B28	13.95	D-Fructose (5TMS)	C_21_H_52_O_6_Si_5_	540	1909
B29	14.18	D-Galactose (5TMS)	C_21_H_52_O_6_Si_5_	540	1930
B30	14.26	D-Glucose (5TMS)	C_22_H_55_NO_6_Si_5_	569	1936
B31	14.32	L-Lysine (4TMS)	C_18_H_46_N_2_O_2_Si_4_	434	1942
B32	14.54	L-Tyrosine (3TMS)	C_18_H_35_NO_3_Si_3_	397	1961
B33	14.64	D-Glucitol (6TMS)	C_24_H_62_O_6_Si_6_	614	1970
B34	14.72	D-Sorbitol (6TMS)	C_24_H_62_O_6_Si_6_	614	1977
B35	15.24	D-Tagatose (6TMS)	C_24_H_61_NO_6_Si_6_	627	2022
B36	15.53	D-Gluconic acid (6TMS)	C_24_H_60_O_7_Si_6_	628	2047
B37	16.12	Palmitic acid (TMS)	C_19_H_40_O_2_Si	328	2098
B38	16.62	Myo-Inositol (6TMS)	C_24_H_60_O_6_Si_6_	612	2142
B39	16.91	Caffeic acid (3TMS)	C_18_H_32_O_4_Si_3_	396	2167
B40	17.16	Oleic acid (TMS)	C_21_H_40_O_2_Si	352	2189
B41	17.81	α-Linolenic acid (TMS)	C_21_H_38_O_2_Si	350	2245
B42	18.09	Stearic acid (TMS)	C_21_H_44_O_2_Si	356	2270
B43	20.23	D-Galacturonic acid (5TMS)	C_21_H_50_O_7_Si_5_	554	2456
B44	22.57	Lactulose (8TMS)	C_36_H_86_O_11_Si_8_	918	2660
B45	23.25	Sucrose (8TMS)	C_36_H_86_O_11_Si_8_	918	2719
B46	23.93	D-Lactose (8TMS)	C_36_H_86_O_11_Si_8_	918	2778
B47	24.19	Maltose (8TMS)	C_36_H_86_O_11_Si_8_	918	2801
B48	25.25	D-Cellobiose (8TMS)	C_36_H_86_O_11_Si_8_	918	2893
B49	26.65	Galactinol (9TMS)	C_38_H_92_O_11_Si_9_	976	3015
B50	29.49	Rosmarinic acid (5TMS)	C_33_H_56_O_8_Si_5_	720	3262
B51	30.95	D-Mannose (8TMS)	C_36_H_86_O_11_Si_8_	918	3389

RT, retention time.

MF, molecular formula.

MW, molecular weight.

RI, retention index.

**Table 4:** Identification of non-volatile metabolites analyzed by UPLC-ESI-Q-TOF-MS/MS.

No.	RT (min)	Adduct ions (m/z)	Molecular ions(m/z)	Fragment ions in MS/MS (m/z)	Molecular formula	Molecular weight	Error (ppm)	Identification	References
C1	4.06	[M+NH_4_]^+^	256.0813	237.9925, 196.9654, 181.0494	C_11_H_10_O_6_	238.0415	-0.99	Acetyloxycaffeic acid	(Ma et al., 2014)
C2	4.44	[M+NH_4_]^+^	344.1340	165.0546, 147.0442, 119.0490	C_15_H_18_O_8_	326.1002	0.14	Coumaric acid-4-*O*-glucoside	(Chaturvedula and Prakash, 2013; Ma et al., 2014)
C3	5.69	[M+H]^+^	209.1535	191.1425, 167.1432, 109.0650	C_11_H_12_O_4_	208.1460	-0.89	Caffeic acid ethyl ester	(Chaturvedula and Prakash, 2013; Ma et al., 2014)
C4	5.98	[M+NH_4_]^+^	406.2073	227.1279, 209.1172, 191.1064, 167.1068, 149.0959, 131.0852	C_18_H_28_O_9_	388.1733	0.08	Tuberonic acid glucoside	(Quirantes-Piné et al., 2010)
C5	6.26	[M+H]^+^	227.1279	191.1071, 163.1112, 149.0964, 131.0855, 107.0857	C_12_H_18_O_4_	226.1205	0.63	Tuberonic acid	(Quirantes-Piné et al., 2010)
C6	6.41	[M+H]^+^	595.1661	577.1559, 457.1138, 379.0818, 325.0710, 295.0601	C_27_H_30_O_15_	594.1589	0.68	Apigenin-7-*O*-dilgucoside	(Yamazaki et al., 2003; Zheng et al., 2020)
C7	8.03	[M+H]^+^	639.1201	463.0880, 287.0554	C_27_H_26_O_18_	638.1129	1.52	Scutellarin-7-*O*-diglucuronide	(Yamazaki et al., 2003; Kaufmann et al., 2016)
C8	9.11	[M+H]^+^	639.1198	463.0876, 287.0553	C_27_H_26_O_18_	638.1126	1.14	Luteolin-7-*O*-diglucuronide	(Meng et al., 2008; He et al., 2015)
C9	10.73	[M+H]^+^	479.0822	303.0501	C_21_H_18_O_13_	478.0749	0.28	Quercetin-3-*O*-glucuronide	(Kaufmann et al., 2016)
C10	10.90	[M+H]^+^	757.1977	595.1453, 449.1088, 287.0558,	C_36_H_36_O_18_	756.1907	0.68	Cis-shisonin	(Yamazaki et al., 2003; He et al., 2015)
C11	11.59	[M+H]^+^	449.1087	287.0555, 153.0181	C_21_H_20_O_11_	448.1013	1.74	Luteoloside*	(Meng et al., 2008; Kaufmann et al., 2016)
C12	11.76	[M+NH4]^+^	374.1449	231.0504, 159.0287, 145.0494, 127.0389	C_15_H_16_O_10_	356.1110	0.83	Caffeic acid-3-*O*-glucuronide	(Zheng et al., 2020)
C13	12.44	[M+H]^+^	623.1252	447.0927, 271.0607, 141.0182	C_27_H_26_O_17_	622.1178	1.36	Apigenin-7-*O*-diglucuronide	(Meng et al., 2008; Kaufmann et al., 2016)
C14	13.15	[M+H]^+^	465.1029	303.0505, 285.0399, 85.0254	C_21_H_20_O_12_	464.0956	0.24	Quercetin-3-*O*-glucoside	(Pereira et al., 2012; Kaufmann et al., 2016)
C15	14.73	[M+H]^+^	757.1975	595.1442, 449.1076, 287.0547	C_36_H_36_O_18_	756.1901	-0.04	Shisonin	(Yamazaki et al., 2003; He et al., 2015)
C16	15.00	[M+H]^+^	463.0877	287.0554	C_21_H_18_O_12_	462.0805	1.49	Scutellarin*	(Yamazaki et al., 2003; Kaufmann et al., 2016)
C17	15.28	[M+H]^+^	463.0876	287.0555	C_21_H_18_O_12_	462.0803	0.98	Luteolin-7-*O-*glucuronide*	(Kaufmann et al., 2016)
C18	15.57	[M+H]^+^	433.1132	271.0604, 153.0181, 85.0282	C_21_H_20_O_10_	432.1059	0.68	Apigenin-7-*O*-glucoside*	(Yamazaki et al., 2003; Kaufmann et al., 2016)
C19	17.39	[M+H]^+^	317.1021	197.0446, 182.0214, 147.0440	C_13_H_16_O_9_	316.0948	0.31	Protocatechuic acid-3-*O*-glucoside	(Yamazaki et al., 2003; Zheng et al., 2020)
C20	18.51	[M+H]^+^	843.1985	595.1451, 535.1078, 287.0547	C_39_H_38_O_21_	842.1912	0.73	Malonyl-shisonin	(Yamazaki et al., 2003; He et al., 2015)
C21	18.88	[M+NH4]^+^	392.2282	195.1380, 177.1271, 149.1328, 135.1169	C_19_H_18_O_8_	374.1944	0.89	Rosmarinic acid methyl ester	(Kaufmann et al., 2016; Zheng et al., 2020)
C22	19.67	[M+NH_4_]^+^	738.2030	523.1245, 343.0818, 181.0496, 163.0390	C_36_H_32_O_16_	720.1693	0.43	Caffeic acid tetramer	(Zheng et al., 2020)
C23	20.11	[M+H]^+^	447.0927	271.0597, 153.0176	C_21_H_18_O_11_	446.0854	1.12	Apigenin-7-*O*-glucuronide*	(Yamazaki et al., 2003; Kaufmann et al., 2016)
C24	21.70	[2M+Na]^+^	743.1578	383.0746, 221.0421, 203.0315, 185.0207	C_18_H_16_O_8_	360.3150	0.5	Rosmarinic acid*	(Zhou et al., 2014; Kaufmann et al., 2016)
C25	29.25	[M+H]^+^	287.0553	241.0497, 153.0183, 135.0439,	C_15_H_10_O_6_	286.0480	0.87	Luteolin*	(Lee et al., 2013; Zhou et al., 2014)
C26	29.40	[M+H]^+^	301.1075	197.0446, 182.0211, 103.0540	C_16_H_12_O_6_	300.1002	1.31	Chrysoeriol	(Lee et al., 2013; Guan et al., 2014)
C27	32.00	[M+H]^+^	271.0601	243.0652, 153.0180, 119.0492	C_15_H_10_O_5_	270.2370	0.14	Apigenin*	(Pereira et al., 2012; Lee et al., 2013)
C28	34.64	[M+H]^+^	609.2814	577.2529, 448.1980, 397.2128,	C_33_H_40_N_2_O_9_	608.2739	-1.9	Reserpine	Internal standard

RT, retention time.

“*”, confirmed with reference substances.

### 3.2 Principal component analysis (PCA) reveals metabolic variation of PLs at different harvest times

**Figure 3:**
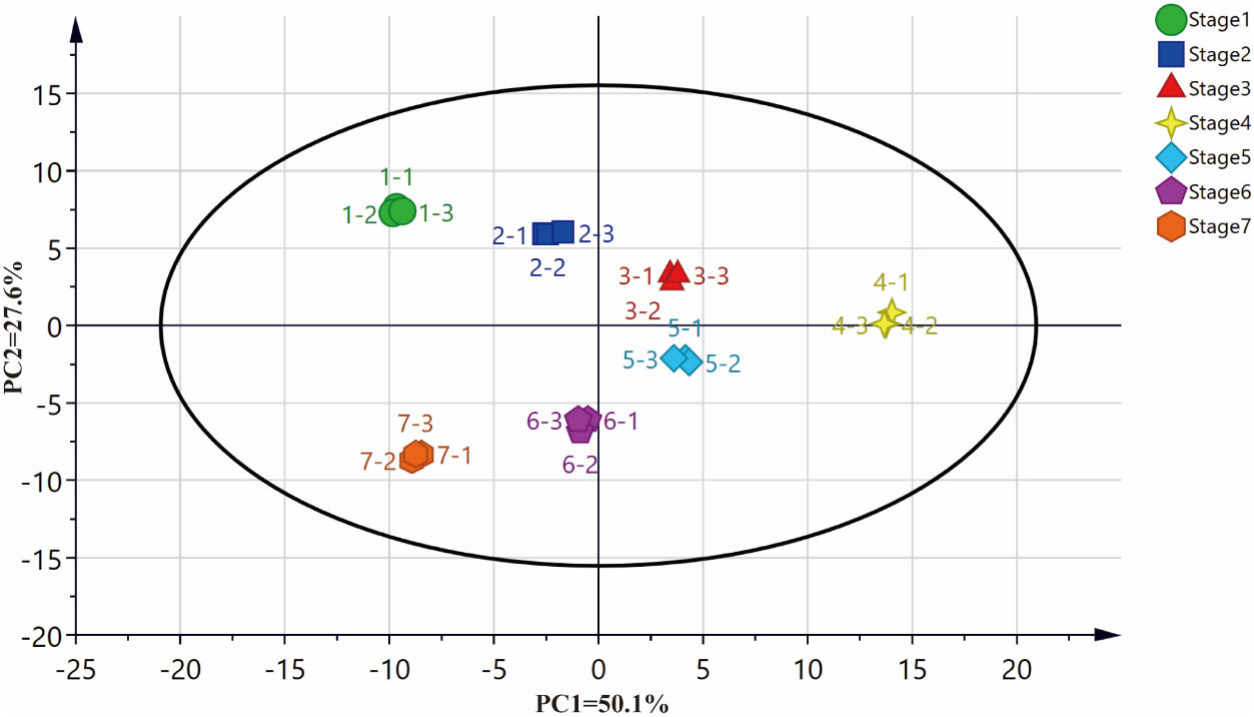
The principal component analysis (PCA) score plots of of PLs samples at different harvesting times.

PCA was carried out for an overview of the dataset. In the PCA plot, three biological replicates of each stage were compactly gathered together (**Figure 3**) while samples at different harvest time were clearly separated indicating metabolomic changes during PLs development. PC1 and PC2 explained 77.7% of the total variance. Samples collected at harvest time 4 were completely separated with samples harvested at other periods on PC1 indicating a special and significant meaning of this harvest period. The loading values of all the metabolites are listed in **Table 5**.

**Table 5:** The PCA loading values and Duncan’s test result of metabolites identified in developing PLs.

No.	Compounds	PC1	PC2	Stage 1	Stage 2	Stage 3	Stage 4	Stage 5	Stage 6	Stage 7
A1	α-Pinene	0.10	-0.04	d	bc	cd	a	ab	bc	cd
A2	Pseudolimonene	0.10	0.00	c	c	b	a	b	b	b
A3	D-limonene	0.08	-0.09	c	bc	bc	a	a	a	b
A4	α-Terpinene	0.09	-0.03	c	d	d	a	bc	b	d
A5	Linalool	0.09	0.08	bc	bcd	d	a	b	cd	e
A6	α-Terpineol	0.11	0.08	de	b	bc	a	cd	e	f
A7	Nerol	0.10	0.07	bcd	bc	b	a	d	cd	e
A8	Perilla ketone	0.05	0.10	bc	a	a	ab	bc	bc	c
A9	Shisool	0.11	-0.09	e	e	c	a	bc	b	d
A10	Perillaldehyde	0.12	0.01	f	b	c	a	d	d	e
A11	γ-Elemene	0.06	-0.12	f	f	e	c	b	a	d
A12	α-Copaene	0.11	-0.08	e	d	d	a	b	c	d
A13	β-Bourbonene	0.11	-0.01	d	d	d	a	b	c	e
A14	β-Elemene	0.10	0.06	c	c	b	a	a	d	d
A15	β-Caryophyllene	0.11	0.06	c	b	bc	a	bc	bc	d
A16	Perillic acid	0.11	0.03	d	d	b	a	c	d	d
A17	α-Humulene	0.10	0.10	c	c	a	a	b	d	f
A18	β-Copaene	0.11	0.05	c	c	b	a	c	b	d
A19	Cis-α-Bergamotene	0.10	0.09	e	b	c	a	d	f	f
A20	Bicyclogermacrene	0.11	0.05	e	c	b	a	d	d	e
A21	α-Farnesene	0.10	-0.07	e	de	cd	a	c	b	cde
A22	Myristicin	0.12	0.00	f	de	b	a	c	d	e
A23	δ-Cadinene	0.10	0.06	c	c	ab	a	a	bc	d
A24	Elemicin	0.11	0.02	cd	cd	bc	a	b	d	d
A25	Nerolidol	0.11	-0.04	e	e	e	a	b	c	d
A26	Espatulenol	0.11	0.08	bc	b	b	a	b	c	d
A27	β-Caryophyllene oxide	0.07	-0.11	d	bc	c	bc	a	ab	bc
A28	α-Patchoulene	0.12	-0.02	f	f	c	a	b	d	e
A29	Isoelemicin	0.11	0.01	cd	cd	bc	a	b	d	cd
A30	Phytyl acetate	0.09	0.05	c	b	b	a	c	c	c
A31	Pentadecanone	0.11	0.01	d	c	d	a	b	d	d
A32	Myristic acid	0.12	0.00	d	c	c	a	b	c	d
A34	Phytol	0.10	0.04	c	c	b	a	d	cd	cd
A36	Heptacosane	0.03	-0.06	c	c	c	c	b	a	d
A37	Squalene	0.12	-0.04	c	c	b	a	b	b	c
A38	Nonacosane	0.09	-0.10	d	c	c	a	bc	ab	bc
A39	1-Heptatriacotanol	0.10	-0.06	d	c	b	a	a	a	cd
A40	Hentriacontane	0.06	-0.14	c	b	b	a	a	a	a
A41	α-Tocopherol	0.11	-0.05	f	d	c	a	a	b	e
A42	Campesterol	0.06	0.11	bc	a	a	a	b	c	c
A43	β-Stigmasterol	0.03	0.12	bc	a	b	bc	bc	c	d
A44	Dotriacontane	0.06	-0.14	d	d	c	b	c	a	bc
A45	γ-Sitosterol	0.07	0.07	d	a	c	b	c	d	d
A46	β-Amyrone	0.06	0.09	e	a	b	c	d	e	e
A47	α-Amyrin	0.02	0.10	cd	a	b	c	c	d	d
B1	Lactic acid	0.11	-0.02	c	b	b	a	b	b	bc
B2	L-Alanine	0.08	-0.13	e	e	d	a	b	c	c
B3&B12	L-Glycine	-0.01	0.17	a	b	c	d	d	e	f
B4	Oxalic acid	0.11	-0.03	d	c	ab	a	bc	c	c
B5	Propanedioic acid	0.09	-0.11	d	c	bc	a	b	b	b
B6	L-Valine	0.11	-0.04	d	c	c	a	b	b	c
B8	L-Leucine	0.12	-0.01	e	d	b	a	b	c	de
B9	Glycerol	0.12	0.00	d	de	c	a	b	d	e
B10	L-Isoleucine	0.12	0.00	e	e	b	a	c	d	e
B11	L-Proline	0.10	-0.10	e	d	c	a	b	bc	c
B13	Glyceric acid	0.11	0.03	c	ab	a	a	a	b	c
B7&14	L-Serine	0.01	0.17	a	ab	b	c	c	d	e
B15	L-Threonine	0.12	-0.04	f	e	b	a	c	d	d
B16	Malic acid	0.11	-0.02	d	b	a	a	b	bc	c
B18	L-Aspartic acid	0.10	0.10	d	b	a	a	c	d	e
B19	γ-Aminobutanoic acid	0.11	-0.01	c	b	a	a	a	b	b
B20	L-Glutamic acid	0.11	-0.01	d	cd	ab	a	bc	bcd	cd
B21	L-Phenylalanine	-0.02	0.17	a	b	c	d	e	f	f
B22&24	L-Asparagine	0.04	0.15	a	a	a	a	b	b	c
B23	Tartaric acid	0.11	0.06	d	b	a	a	c	c	d
B25	Xylitol	0.01	0.17	a	b	b	b	c	d	d
B26	L-Glutamine	0.11	-0.08	f	e	b	a	c	c	d
B27	Citric acid	0.11	0.04	d	ab	ab	a	b	c	cd
B28	D-Fructose	0.02	-0.16	f	e	d	cd	c	b	a
B29	D-Galactose	-0.01	-0.17	e	d	c	c	b	a	a
B30	D-Glucose	0.04	-0.16	e	d	c	b	b	a	a
B31	L-Lysine	-0.03	0.17	a	b	c	d	e	f	f
B32	L-Tyrosine	0.00	0.17	a	a	bc	b	c	d	e
B33	D-Glucitol	-0.02	0.17	a	a	b	bc	c	d	d
B34	D-Sorbitol	-0.07	-0.15	d	d	e	e	c	b	a
B35	D-Tagatose	-0.02	-0.15	c	c	c	b	b	b	a
B36	D-Gluconic acid	-0.04	-0.16	f	e	f	d	c	b	a
B37	Palmitic acid	0.10	0.07	c	b	b	a	c	c	c
B38	Myo-Inositol	-0.04	0.15	a	b	bc	cd	cd	d	d
B39	Caffeic acid	0.11	-0.08	e	d	b	a	a	b	c
B40	Oleic acid	0.12	-0.03	f	f	b	a	c	d	e
B41	α-Linolenic acid	0.10	0.05	c	cd	b	a	c	de	e
B42	Stearic acid	0.12	0.01	d	cd	b	a	bc	cd	d
B43	D-Galacturonic acid	-0.04	0.13	a	b	b	b	bc	c	d
B44	Lactulose	-0.03	-0.17	f	ef	de	d	c	b	a
B45	Sucrose	-0.03	-0.16	d	cd	cd	c	c	b	a
B47	Maltose	-0.07	-0.13	c	c	c	c	c	b	a
B46	D-Lactose	-0.04	-0.16	e	d	d	d	c	b	a
B48	D-Cellobiose	-0.03	-0.13	c	b	b	b	b	b	a
B49	Galactinol	-0.03	0.16	a	b	b	c	d	d	e
B51	D-Mannose	-0.03	-0.14	e	c	cd	d	cd	b	a
C1	Acetyloxycaffeic acid	0.06	-0.11	e	e	c	b	d	a	c
C2	Coumaric acid-4- *O-* glucoside	0.11	-0.02	c	b	a	a	a	a	d
C3	Caffeic acid ethyl ester	0.00	-0.15	f	g	d	c	e	a	b
C4	Tuberonic acid glucoside	0.12	-0.01	f	e	c	a	b	d	g
C5	Tuberonic acid	0.12	0.00	f	e	b	a	c	d	f
C6	Apigenin-7-*O*-dilgucoside	0.12	0.06	e	c	b	a	c	d	f
C7	Scutellarin-7-*O*-diglucuronide	0.11	-0.01	d	e	a	a	b	c	e
C8	Luteolin-7-*O*-diglucuronide	0.11	0.02	bc	b	b	a	b	c	c
C9	Quercetin-3-*O*-glucuronide	0.12	0.02	f	d	b	a	c	d	e
C10	Cis-shisonin	0.08	-0.02	d	d	d	b	a	c	e
C11	Luteoloside	0.12	-0.05	d	c	c	a	b	c	c
C12	Caffeic acid-3-*O*-glucuronide	0.06	-0.13	f	f	d	b	e	a	c
C13	Apigenin-7-*O*-diglucuronide	0.13	0.00	d	c	c	a	b	c	d
C14	Quercetin-3-*O*-glucoside	0.11	0.05	d	b	a	a	a	c	e
C15	Shisonin	0.04	-0.07	e	d	cd	c	a	b	cd
C16	Scutellarin	0.12	-0.01	d	c	b	a	b	b	d
C17	Luteolin-7-*O*-glucuronide	0.12	-0.01	f	d	ab	a	bc	cd	e
C18	Apigenin-7-*O*-glucoside	0.12	-0.03	e	c	b	a	b	c	d
C19	Protocatechuic acid-3-*O*-glucoside	0.10	0.08	e	a	a	a	b	c	d
C20	Malonyl-shisonin	0.06	-0.07	e	d	cd	b	a	b	c
C21	Rosmarinic acid methyl ester	0.06	0.06	c	d	a	b	e	c	f
C22	Caffeic acid tetramer	0.11	-0.05	f	d	d	a	b	c	e
C23	Apigenin-7-*O*-glucuronide	0.12	-0.03	e	c	b	a	b	b	d
C24	Rosmarinic acid	0.11	-0.09	e	d	b	a	b	b	c
C25	Luteolin	0.11	0.06	d	c	b	a	a	cd	e
C29	Chrysoeriol	0.11	0.08	e	c	b	a	d	e	f
C27	Apigenin	0.12	0.05	d	c	c	a	b	d	e

a, b, c, d, e, f indicated significant levels according to Duncan’s test (p < 0.05).

### 3.3 Clustering analysis reveals dynamic patterns of metabolites in PLs during developmental process

**Figure 4:**
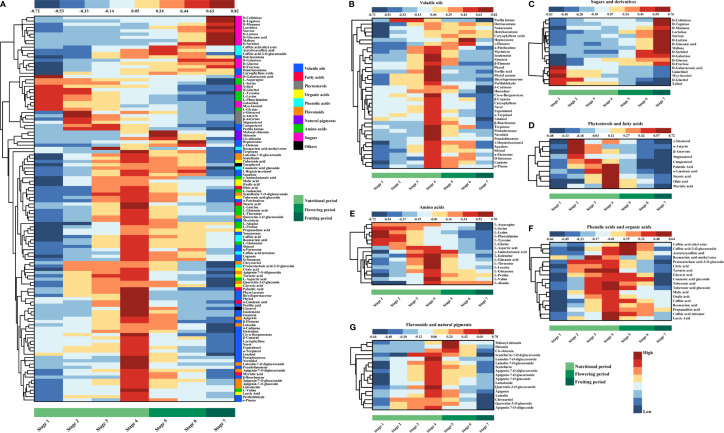
Metabolome dynamics of developing PLs. **(A)** Overview of the metabolite dynamics with clustering heat map. **(B–G)** Present the dynamics of volatile oils, sugars, phytosterols and fatty acids, amino acids, phenolic acids and organic acids, derivatives, flavonoids and anthocyanins.

To observe the dynamic changes of metabolites in different harvest periods in a more intuitive manner, a heatmap of the 118 different metabolites was obtained (**Figure 4A**).

#### 3.3.1 Dynamic patterns of volatile compounds

Volatile oil is a very important and widely studied class of metabolites in perilla. They showed bioactivities such as antibacterial, antiviral, anti-inflammatory, anticarcinogenic, antioxidant, etc (Raut and Karuppayil, 2014). In most flowering plants, the production and emission of volatile metabolites are developmentally regulated and show similar developmental characteristics. Normally, volatile oil accumulates in the early developmental stage when fruits are not mature or before the flowers are ready for pollination. Then a release of volatile components to attract pollinators might cause a decrease of volatile compounds in the early stage of flowering (Dudareva et al., 2000; Dudareva et al., 2013). In the present study, most of volatile oil compounds showed highest level at stage 4 which was pre anthesis period (**Figure 4B**). Only heptacosane and γ-elemene showed the highest level at stage 6 which was a stage before fruiting period (**Figure 4B**). The dynamic patterns of volatile compounds indicated their crucial function in plant pollination and reproduction.

#### 3.3.2 Sugars and derivatives

During photosynthesis, all kinds of carbon is fixed in the forms of sugars and sugar derivatives (Smeekens and Hellmann, 2014; Sakr et al., 2018) Sugars help plants store energy and play essential roles in signalling pathways of plant growth and development. In this study, the main sugars in PLs are D-fructose, D-glucose and sucrose. They accumulated constantly during the developmental process of PLs and were with highest levels in fruiting phase (**Figure 4C**). Most of the sugars and sugar derivatives showed similar dynamic patterns as them (**Figure 4C**). Only five sugar derivatives (xylitol, D-glucitol, myo-inositol, galactinol and galacturonic acid) changed differently, with higher content at early developmental stage and decreased throughout the development process (**Figure 4C**). Accumulation of sugar content during plant development was also observed in Cichorium spinosum (Petropoulos et al., 2018).

#### 3.3.3 Phytosterols and fatty acids

The sterol composition of plants is complex and diverse. The main membrane sterols in higher plants are β-sitosterol, stigmasterol and campesterol (Ruan, 2014). Sterols are not only signal and regulatory molecules involved in plant growth and development, but also play key roles in cell proliferation and differentiation (Guo et al., 1995; Moreau et al., 2018). In this study, all phytosterols were showed the highest level at stage 2, and decreased gradually (γ-sitosterol, β-amyrone, α-amyrin, stigmasterol, campesterol) (**Figure 4D**). This trend may be due to the vigorous metabolism of cells in the nutritional stage.

Fatty acids and lipids provide structural integrity and energy for various metabolic processes (Lim et al., 2017). The predominant fatty acids detected in PLs were palmitic acid, oleic acid and α-linolenic acid which increased pre anthesis period and declined afterwards (**Figure 4D**). Oleic acid and α-linolenic acid are essential unsaturated fatty acids (UFAs) and recommended for consumption for their multiple health benefits, such as anti-obesity (Fan et al., 2020), cardioprotection (Russell et al., 2020), anti-diabetes (Canetti et al., 2014), anti-inflammation (Wang et al., 2020), anti-cancer (Schiessel et al., 2015), neuroprotection (Kumari et al., 2019) and so on. Intake of α-linolenic acid rich *P. frutescens* leaf powder in Japanese adults showed some cardiovascular protective effects (Hashimoto et al., 2020). Considering the health benefits of these unsaturated fatty acids, stage 4, the pre anthesis period would be suitable harvest time for ensuring high content of these UFAs in perilla leaves.

#### 3.3.4 Amino acids

Amino acids are not only important components for plants to complete their life cycle activities (Paulusma et al., 2022), but also essential nutrients for humans and other animals. PLs are rich in amino acids. Amino acids in PLs showed two distinct dynamic patterns during PLs development. Some amino acids were with higher content at early stages and decreased throughout the developmental process, such as L-serine, L-lysine, L-phenylalanine, L-tyrosine, L-glycine (**Figure 4E**). Other amino acids were showed the highest level at stage 4, and decreased afterwards, such as L-aspartic acid, L-isoleucine, L-threonine, L-leucine, L-glutamine, L-proline, L-valine, L-alanine, etc (**Figure 4E**). Free amino acids could elicit complex gustatory sensation (Kawai et al., 2012), especially the taste of umami. They can bring fresh and brisk tastes to PLs and participate in the formation of aroma substances (Lee et al., 2019). With the maturity and senescence of leaves, there may be two reasons for the decrease of amino acids. First, amino acids might be involved in the synthesis of storage proteins. Second, the complete oxidation of amino acids produces the energy required to meet the special needs of certain organs, such as stressed leaves or roots. The molecular mechanism of regulation of amino acid catabolism in plants is complex and unclear so far (Hildebrandt et al., 2015). Considering the nutritional value and gustatory sensation of amino acids, it would be appropriate to harvest perilla leaves before the pre anthesis period.

#### 3.3.4 Phenolic acids and organic acids

Phenolic acids have various pharmacological activities, such as anti-inflammatory, anti-anxiety, and anti-depressive activities (Tinikul et al., 2018; Deguchi and Ito, 2020). Some of them are connected to the polymer of the cell wall through covalent bonds, which is crucial to the process of plant immune mechanism (Stuper-Szablewska and Perkowski, 2019). The predominant phenolic acids detected in PLs were rosmarinic acid and caffeic acid, which showed highest level at stage 4 (**Figure 4F**).

Organic acids are the intermediate products of cell metabolic tricarboxylic acid (TCA) cycle (Xiao and Wu, 2014). Many environmental stresses stimulate the biosynthesis and release of organic acids. For example, plants secrete organic acids in root exudates to mobilize phosphorus in deficient soil (Panchal et al., 2021). The main organic acids in PLs are lactic acid, malic acid, tartaric acid and citric acid. They also increased at early stages, showed highest level at around pre anthesis period and decreased afterwards (**Figure 4F**). Organic acids contribute to the sourness and fruity taste of plants, while inhibit the bitterness taste (Wang et al., 2021). Therefore, considering the high content of these compounds in PLs at the stage 4, alternative uses for food or pharmaceutical can be proposed.

#### 3.3.5 Flavonoids and natural pigments

Flavonoids play an important role in plant development and defense, have the ability to scavenge reactive oxygen species (ROS) and protect plants against damage from biotic and abiotic stresses (Iwashina, 2003; Pourcel et al., 2007). During perilla leaves development, the detected flavonoids presented an unanimous changing pattern. All the flavonoids accumulated pre anthesis period and showed the highest level at stage 4 (**Figure 4G**). Previous studies reported that flavonoids have many biological functions such as anti-inflammatory, anti-oxidative, anti-diabetic, and anti-hypertensive activities (Kawser Hossain et al., 2016; Jiang et al., 2020).

The color of fruits and flowers is crucial in plant ecology, can attract pollinators and seed-dispersal organisms (Grotewold, 2006). The molecular signals that induce pigment biosynthesis during pollination are unclear, but light plays a central role (Farzad et al., 2002). Natural pigments from PLs have exhibited a wide range of bioactive properties including antioxidant effects, anti-inflammatory effects, etc (Chang et al., 2005; Wang and Stoner, 2008; Lila et al., 2016). Natural pigments detected in PLs including shisonin and its derivatives. They showed the highest level at stage 5 (vigorous flowering period) (**Figure 4G**). According to this result, if the targeted metabolites are these pigments, it is better to harvest PLs during flowering period.

## 4 Conclusion

In this study, our results showed the advantages of applying an integrated LC-MS and GC-MS metabolomic platforms the evaluation of optimal harvesting period for plants. We employed metabolomic analysis to clarified the evolutionary trajectories and dynamic changes of volatile oil compounds, sugars, flavonoids, amino acids, organic acids, etc. The results of this study provide a theoretical basis for the development of PLs and offer data support for the optimal harvesting period of PLs. Considering the content of most of the nutrients and bioactive components, pre anthesis period is a suitable harvest time for PLs.

## Data availability statement

The original contributions presented in the study are included in the article/supplementary material. Further inquiries can be directed to the corresponding author.

## Author contributions

LW and YZ conceived and designed the experiments. JC performed the experiments. JC, GY, and AY analyzed the data. JC wrote the manuscript. LW, YZ and LG revised and edited the manuscript. All authors have read and agreed to the published version of the manuscript.

## Funding

This research was funded by Natural Science Foundation of Hebei Province (C2020423047); Research Foundation of Hebei Provincial Administration of Traditional Chinese Medicine (2019083); The Innovation Team of Hebei Province Modern Agricultural Industry Technology System (HBCT2018060205).

## Acknowledgments

We would like to thank Prof. Chunxiu Wen and her team for providing us the plant materials. We would like to thank the gardeners for their great maintenance of the perilla. We would like to thank all the members in Traditional Chinese Medicine Processing Technology Innovation Center of Hebei Province for fruitful discussions.

## Conflict of interest

The authors declare that the research was conducted in the absence of any commercial or financial relationships that could be construed as a potential conflict of interest.

## Publisher’s note

All claims expressed in this article are solely those of the authors and do not necessarily represent those of their affiliated organizations, or those of the publisher, the editors and the reviewers. Any product that may be evaluated in this article, or claim that may be made by its manufacturer, is not guaranteed or endorsed by the publisher.
